# Owning your mistakes

**DOI:** 10.7554/eLife.11628

**Published:** 2015-10-22

**Authors:** Eve Marder

**Affiliations:** Department of Biology and the Volen National Center for Complex Systems, Brandeis University, Waltham, United Statesmarder@brandeis.edu

**Keywords:** living science, scientific excellence, scientific publishing, reproducibility, replication

## Abstract

Most scientists admit to their errors but, as **Eve Marder** explains, the scientific community as a whole needs to rethink the way it recognizes achievement.

I confess that I often listen to sports radio during my 20 min commute to and from work. About a year ago, I first heard the phrase “owning your mistakes” on sports radio, and that term has stayed with me since then. Listening to sports radio gives one insight into the moral compass of a swath of the American public (and is a welcome relief from the litany of violence, disaster and true evil that permeates the real news from all over the globe). The sportscasters and the members of the public who phone in are forgiving of people who have made mistakes and are willing to “own” them. For example, the coach who called the wrong play, or the player who said something dumb when interviewed, are easily forgiven when they “own their mistakes”.

In contrast, the sportscasters and the public are less forgiving of those who make mistakes and then deny them, and they are especially intolerant of repeated patterns of bad behavior. The concept of fairness is important to the listeners of sports radio, and they are very aware that the world of professional sports is populated by individuals who were given the gift of unusual skills: this means that they are completely unsympathetic to those who squander their gifts by not working hard and taking their jobs seriously.

I see many parallels between the world of science and the world of sports. All of us who attempt to do science that breaks new ground are likely to make honest mistakes. Naturally we will try to make as few mistakes as possible, but it is inevitable that when we are working at the frontiers of knowledge, we will not always know which control experiments are necessary. Or some as-yet-unknown factor will turn out to play an unforeseen role in the processes we are studying. There are an infinite number of ways that honest errors can creep into our work. Or, as recently happened to a colleague of mine, something that has worked for 20 years will just stop working, for unknown reasons.

All of us who attempt to do science that breaks new ground are likely to make honest mistakes.

In another example, one of my PhD students recently failed to replicate some of our previously published observations, for reasons we still don't fully understand. This was obviously painful, but we published the most complete description of the new data and our best assessment of what could be responsible for the discrepancy (including the possibility that our wild-caught crab populations are affected by climate change). Ironically, the first version of the manuscript was rejected (not by *eLife*) because a reviewer said we shouldn't be allowed to publish something that disagreed with our own prior observations!

There are many reasons why laboratories or investigators can generate data that appear to be discrepant. Unlike analytic solutions in math, biology has always benefitted from some experiments that require ‘good hands’, or the virtuosity needed to do an experiment with enough care and skill to generate stunning images or really clean data. We know that laboratory work includes some artistry, and that artistry is difficult to explain, teach or describe in a methods section. Each investigator consciously or unconsciously makes modifications in ‘lab lore’, often without realizing that a small tweak that does not seem to change anything could influence the experiment under different conditions.

And even the most careful proof reader can miss the absence of a negative sign in text he or she has written and read many times, so inadvertent mistakes in published papers are an unfortunate feature of the scientific literature. In the latter case, these mistakes are usually noticed by someone ‘out there’ and can be simply acknowledged and fixed. Some errors are self-correcting, such as when a new student innocently applies dopamine made in distilled water rather than saline, and the preparation dies. Others are harder to spot, especially as the tradition of maintaining meticulous laboratory notebooks (a practice that helps in the detection of errors) is being eroded.

As Lisa Feldman Barrett, a professor of psychology at Northeastern University, explained in the *New York Times* recently, honestly reported failures to replicate can bring important insight into the unknown constraints and conditions that influence phenomena, and therefore provide a path to new insight and discovery. I take great comfort when two or more laboratories (either competitors or collaborators) do approximately the same experiments and produce similar, if not identical, results. To the extent to which a finding is important, it is that much more important to know that competing laboratories obtain essentially the same conclusions. For this reason, I have never been able to understand the logic of journals that refuse to publish a finding if it was ‘scooped’ by a few weeks or months.

When we turn the pursuit of truth into a race to be first, we encourage sloppiness and increase the likelihood of error (Marder, 2015; this essay also discusses some of the differences between science and sports). So until we embrace the notion that some amount of independent replication deserves the same accolades as the first publication of an important finding, we are *de facto* creating the temptation to cut corners. In the spirit of owning our errors, I suggest that by unduly rewarding the group with the first report of an important finding, we have collectively created an atmosphere with disincentives for ‘getting it right’ if doing so means losing the race to be first.

Mistakes also play an important role in the arts. “The influence of a mistake in a drawing is a wonderful and unexpected thing”, says Ben Marder, the artist who illustrates all my *Living Science* articles. “As an artist, mistakes are so very important. A mistake acts as a bridge from what's in our mind to what is on the paper, and it takes us in new directions that we could not anticipate”.

While we should individually own and correct our honest errors, we should collectively relinquish the focus on prestige publications and honors (such as big grants, prizes and membership of elite academies and societies) that increases the likelihood of corners being cut and avoidable errors being made. In American football, the winner is the team that scores the most points, and instant replay helps referees and umpires to get it right most of the time. In science, scores result from often elusive and subjective judgments. And while sports fans argue endlessly about who should be named a Hall of Fame quarterback, their criteria are far more straightforward than those that govern the award of honors in science, and they do not distort the sports concerned. Sport needs winners and losers—the same should not be true in science.

**Figure 1:**
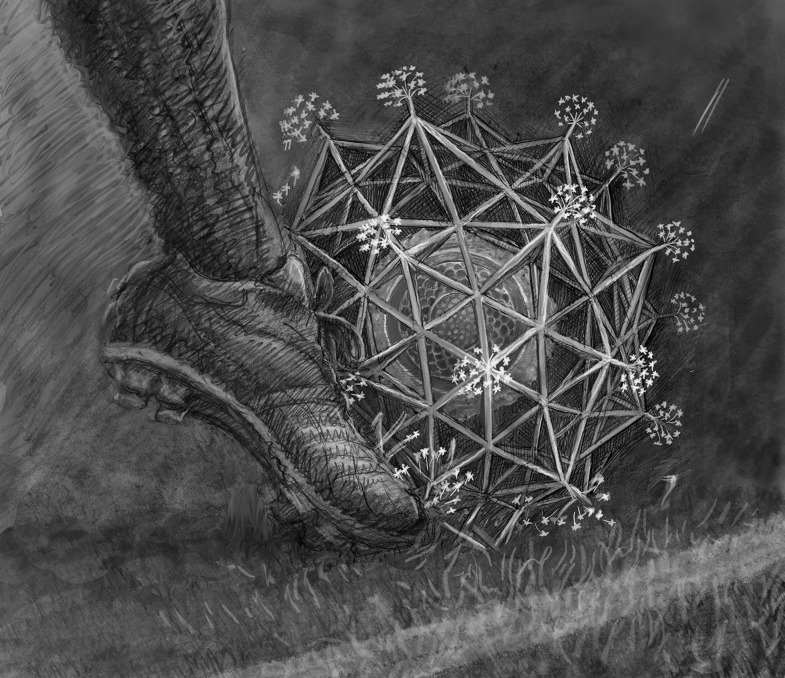
There are many parallels between science and sports, including the need to acknowledge mistakes.

Illustration: Ben Marder.
